# Glimmer of Hope in the Fight to Reduce Human Immunodeficiency Virus Transmission in Pregnancy

**DOI:** 10.1155/S106474499400030X

**Published:** 1994

**Authors:** Daniel V. Landers

**Affiliations:** Department of Obstetrics, Gynecology, and Reproductive Sciences University of California at San Francisco San Francisco California USA


Infectious Diseases in Obstetrics and Gynecology 2:1–2 (1994)

© 1994 Wiley-Liss, Inc.

### Glimmer of Hope in the Fight to Reduce Human Immunodeficiency Virus Transmission in Pregnancy

Women and children presently comprise the group of human immunodeficiency virus (HIV) individuals with the highest rate of newly identified HIV infection. Virtually all newly diagnosed cases of HIV among children occur by vertical transmission of the virus from mother to infant. This area of HIV research has yielded the most promising results in reducing the risk of HIV transmission. Recently, the National Institutes of Health (NIH)-sponsored AIDS Clinical Trials Group (ACTG) halted enrollment midway through ACTG Study 076 due to the overwhelming significance of zidovudine treatment in reducing the rate of vertical transmission (8.3% in the treatment group vs. 25.5% in the placebo group).[1] This trial was restricted to women with CD4 lymphocyte counts >200 cells/mm$^3$ who had not previously received zidovudine. Other clinical trials aimed at reducing vertical transmission are currently underway in women with lower CD4 counts or symptomatic HIV disease.

The report in this issue by Robinson and Fleischer, "Prenatal Human Immunodeficiency Virus Testing and Patient Management by Obstetricians in a High Seroprevalence Community," highlights a crucial challenge facing the providers of women's health care today. The challenge is to identify and counsel all HIV-infected women who might benefit from zidovudine and various other treatments being developed to reduce vertical transmission. The study by Robinson and Fleischer indicates that, even in a high-seroprevalence community, many obstetricians do not routinely offer HIV testing to women without identifiable risk factors. About 40% of the obstetricians responding did not offer HIV testing to all new obstetric patients. Furthermore, as many as 15% of obstetricians responded that they do not routinely even inquire about potential risk factors. Although many HIV-infected women have no identifiable risk factors, their partners may have significant risk factors of which these women are unaware. This fact further emphasizes the need for universal screening during pregnancy.

Obstetricians have the opportunity to positively impact the lives of many HIV-infected women. The first step is to actively offer screening to women. Every woman deserves the opportunity to be screened for a condition that may significantly alter her life and that of her unborn baby, especially as modalities to alter the outcome are becoming available. The goal is not to coerce women into unwanted testing but to provide the information and potential benefits that will facilitate informed choices. Our responsibility goes further than just offering testing and counseling. Once identified, an HIV-positive woman must be provided quality, comprehensive, nonjudgmental care in a supportive environment if pregnancy and HIV outcomes are to be optimized. If the practitioner feels unqualified to provide quality care, then appropriate consultation and referrals may be necessary. Whether providing care directly or through consultations or referral, we as obstetricians have a responsibility to the women we serve to provide optimal care regardless of their HIV status. Neither can we justify in ourselves nor accept in our colleagues

Received July 18, 1994
Accepted July 21, 1994

Editorial judgmental attitudes, refusal to provide services, or other forms of discriminatory behavior that amount to patient abandonment. We must never lose sight of the fact that the enemy is HIV disease, not its victims. Ultimately, we all will suffer from the toll and consequences of HIV infection.

*Daniel V. Landers, M.D.*
*Department of Obstetrics, Gynecology, and Reproductive Sciences*
*University of California at San Francisco*
*San Francisco, California*

### REFERENCES

1. Centers for Disease Control: Zidovudine for the prevention of HIV transmission from mother to infant. MMWR 43(16):185–187, 1994.

